# Effect of Particle Sizes and Contents of Surface Pre-Reacted Glass Ionomer Filler on Mechanical Properties of Auto-Polymerizing Resin

**DOI:** 10.3390/dj11030072

**Published:** 2023-03-03

**Authors:** Naoyuki Kaga, Sho Morita, Yuichiro Yamaguchi, Takashi Matsuura

**Affiliations:** 1 Section of Fixed Prosthodontics, Department of Oral Rehabilitation, Fukuoka Dental College, Fukuoka 814-0193, Japan; 2 Oral Medicine Research Center, Fukuoka Dental College, Fukuoka 814-0193, Japan

**Keywords:** auto-polymerizing resin, flexural modulus, flexural strength, S-PRG filler, three-point bending test, Vickers hardness

## Abstract

Herein, the mechanical properties of an auto-polymerizing resin incorporated with a surface pre-reacted glass ionomer (S-PRG) filler were evaluated. For this, S-PRG fillers with particle sizes of 1 μm (S-PRG-1) and 3 μm (S-PRG-3) were mixed at 10, 20, 30, and 40 wt% to prepare experimental resin powders. The powders and a liquid (powder/liquid ratio = 1.0 g/0.5 mL) were kneaded and filled into a silicone mold to obtain rectangular specimens. The flexural strength and modulus (*n* = 12) were recorded via a three-point bending test. The flexural strengths of S-PRG-1 at 10 wt% (62.14 MPa) and S-PRG-3 at 10 and 20 wt% (68.68 and 62.70 MPa, respectively) were adequate (>60 MPa). The flexural modulus of the S-PRG-3-containing specimen was significantly higher than that of the S-PRG-1-containing specimen. Scanning electron microscopy observations of the specimen fracture surfaces after bending revealed that the S-PRG fillers were tightly embedded and scattered in the resin matrix. The Vickers hardness increased with an increasing filler content and size. The Vickers hardness of S-PRG-3 (14.86–15.48 HV) was higher than that of S-PRG-1 (13.48–14.97 HV). Thus, the particle size and content of the S-PRG filler affect the mechanical properties of the experimental auto-polymerizing resin.

## 1. Introduction

Auto-polymerizing resin (APR) has been used for denture repair or provisional restoration. Usually, denture base fractures are caused by periodic fatigue of the denture base or the clasp arms of removable partial dentures, which are loaded under bending fatigue resulting from repeated mastication forces [1]. Fractured dentures and their repair are common in routine clinical practice. Fractured denture bases are repaired because remaking the dental prosthesis is expensive and time-consuming. Denture repair resins should be strong enough, have good dimensional stability, be simple to use, and be reasonably priced. The repaired denture bases may cause similar fractures at the repaired interface. The flexural strength was lower in the denture base’s repaired area processed by different methods and repair materials than in the sound denture base material [2]. If the repaired denture has sufficient fracture resistance against chewing forces, it provides stability for repairing the denture or clasp fractures and deformations [3]. However, the material used for provisional restoration must have adequate rigidity and fracture toughness to withstand complex mastication forces. APRs are among the most commonly used materials for this purpose. Notably, the fabrication of faultless fixed provisional restorations is crucial to ensure gingival health, pulp protection, the proper functioning of final prostheses, and to reduce dental abutment migration [4].

Typically, biofilm accumulation due to oral microbacteria and regular diets may result in oral diseases such as caries, periodontitis, and denture stomatitis. Moreover, the elderly are also at risk of developing systemic diseases such as infectious endocarditis, aspiration pneumonia, and gastrointestinal infections [5]. Furthermore, biofilms adhere to denture surfaces if the bacteria and fungi colonizing the dentures are not removed [6]. Therefore, preventive and systematic strategies must be formulated to reduce oral health problems among denture wearers. In addition, the long-term use of provisional restorations may facilitate bacterial adhesion and cause inflammation of the marginal gingiva. A promising approach to address these issues is the development of a material capable of automatically activating specific functions by releasing chemical substances or ions when dentures are placed in the oral cavity. The release of such chemical substances can inhibit bacterial and fungal growth and thereby prevent the formation of plaque and biofilms on denture surfaces [7].

To strengthen such defenses and improve oral health, various approaches have been developed to fabricate functional biomaterials that can serve as alternative materials in restorative and prosthodontic dentistry. Previous studies have presented the surface pre-reacted glass ionomer (S-PRG) filler as a bio-functional additive with ion release and recharge abilities to exert bioactive effects. The function of the S-PRG filler with the release of Al, B, F, Na, Si, and Sr ions provides a prospect for clinical applications [8]. The slowly released ions cause bio-actions, such as acid buffering, the inhibition of enamel demineralization [9,10,11,12], antibacterial activity [13,14], enhanced bone formation [15], and Candida biofilm reduction [16].

In some recent studies, fillers have been mixed with materials for dental prostheses, and their mechanical properties have been investigated [17]. Notably, the polymethyl methacrylate (PMMA) resin has been widely used as a material for dentures and temporary crowns. Three-dimensional printing has also been used in the dental field, and new dental materials, such as photopolymers, have been employed [18]. However, the mechanical properties of photopolymers alone are inadequate; hence, satisfying the requirements of ISO standards is difficult. Interestingly, the addition of zirconia fillers to PMMA resins is known to alter the mechanical properties [19]. However, the aforementioned fillers affect only the mechanical properties.

This study investigates the S-PRG filler, which is a cutting-edge filler that releases bioactive components and is used with various dental materials [20]. To ensure a sustained ion release and antibacterial effects in prosthetic materials, the S-PRG filler is included in the acrylic resin used for denture base materials [21] and the tissue conditioner [22] of the mucosal surface conditioning material. Mukai et al. reported that denture base resins containing S-PRG fillers can inhibit dentin demineralization, which may help prevent root caries of tooth abutment [21]. In another study, a bioactive glass that gradually released fluoride ions was added to the PMMA resin and investigated as a caries-prevention material [23]. However, the effect of the release of fluoride ions weakened gradually. In other words, the abovementioned release effect may be temporary even if bioactive glass fillers or S-PRG fillers are included in the prosthetic materials used for long-term treatment.

Further, APRs have been commonly used in prosthodontics for the fabrication of denture bases, denture repair resin, occlusal splints, and temporary crowns [4]. Furthermore, abutment teeth of removable partial dentures and temporary crowns may increase the caries risk. Therefore, it is clinically significant to develop prosthetic materials for denture repair resins and temporary crowns close to the abutment teeth with root surface caries prevention and gingivitis prevention. Since the development of S-PRG fillers, many studies have attempted to find out their superior functions and actions as biomaterials [9,10,11,12,13,14,15,16]. The characteristics of such fillers can be leveraged to develop provisional materials for the oral cavity by incorporating S-PRG fillers into APRs. Generally, a clinically durable mechanical strength value is required when S-PRG fillers are used in APRs. Hence, an investigation of their mechanical properties is essential.

Denture repair material and provisional restorations employ APRs, whereas denture base materials employ heat-polymerizing PMMA resins. S-PRG fillers have been used in preventive and restorative dental products; however, their use with prosthodontic PMMA materials is yet to be reported. The sizes, shapes, and concentrations of the filler particles are known to affect the mechanical strength of PMMA [24]. Accordingly, previous studies have evaluated the effects of the particle size and concentration on flexural strength, flexural modulus, and fracture surfaces. For this, nanoparticles such as zirconium dioxide (ZrO_2_) and aluminum oxide (Al_2_O_3_) have been used as fillers in PMMA [24,25,26]. However, the mechanical properties of APRs incorporating S-PRG fillers with different particle sizes and concentrations are yet to be investigated. Hence, in this study, we investigate, for the first time, the effects of different particle sizes and concentrations of S-PRG fillers on the mechanical properties of APRs. Moreover, we evaluate the correlation between the mechanical properties of APRs and the S-PRG filler concentration and particle size.

Overall, this study aims to assess the effects of two particle sizes, i.e., 1 and 3 μm, and different concentrations of S-PRG fillers on the mechanical properties of APRs. The null hypothesis is that an APR incorporated with an S-PRG filler does not possess mechanical properties adequate for clinical requirements.

## 2. Materials and Methods

### 2.1. Preparation of Specimens

For our analysis, APR powder (self-curing acrylic resin, polymers: Provinice, 3S, pink, lot no. 121804, Shofu Inc., Kyoto, Japan) was mixed with S-PRG fillers with average particle sizes of approximately 1 μm (S-PRG-1) and 3 μm (S-PRG-3) (lot no. PRG1-R3 and lot no. BOG3-65, provided by Shofu Inc. Kyoto, Japan). For the preparation of specimens, the resin powder and each S-PRG filler type at 10, 20, 30, and 40 wt% were mixed using a vortex mixer (NS-80, AS ONE Inc., Osaka, Japan) for 10 min, according to the procedure described by Mukai et al. with some modifications [21]. The same APR powder with a 0 wt% S-PRG filler was used as the control group.

For the three-point bending test, a rectangular specimen with dimensions of 25 mm × 2 mm × 2 mm was prepared according to the ISO standards [27]. Further, for the Vickers hardness test, a disk-shaped specimen with a diameter of 15 mm and a thickness of 1 mm was prepared. The experimental resin powder and a liquid (monomer: lot no. 012041 Shofu Inc., Kyoto, Japan; powder/liquid ratio of 1.0 g/0.5 mL) were kneaded according to the manufacturer’s instructions at room temperature (24 ± 1 °C) to achieve a doughy consistency, and the mixture was filled into silicone molds. The specimens were then covered with a polyethylene sheet, compressed using a metal plate, and maintained at room temperature for 30 min. The specimens were then demolded and immersed in distilled water at 37 ± 1 °C for 24 ± 2 h before testing.

### 2.2. Bending Test

As stated, a bending test was performed to determine the flexural strength and modulus. A three-point bending test jig was used to support the specimen on two parallel rods spaced 20 mm apart. The specimen was loaded centrally at a cross-head speed of 1.0 mm/min using a universal testing machine (Autograph AGS-J, Shimadzu Co., Kyoto, Japan) until failure (*n* = 12).

The specimen was loaded centrally at a cross-head speed of 1.0 mm/min using a universal testing machine (Autograph AGS-J, Shimadzu Co., Kyoto, Japan) until failure occurred (*n* = 12).

Flexural strengths (*FS*, MPa) of the specimens were calculated using Equation (1):

*FS* = 3*Fl*/2*bh*^2^ (1)

where *F* denotes the maximum applied load (N), l denotes the support span distance (20.0 mm), and *b* and *h*, respectively, denote the specimen width (mm) and height (mm) prior to testing.

The flexural moduli (*FM*, GPa) of the specimens were calculated using Equation (2):

*FM* = *F*_1_*l*^3^/4*bh*^3^*d* (2)

where *F*_1_ denotes the load (N) at a point on the straight-line portion of the flexural load–defection curve, and *d* denotes the deflection (mm) at load *F*_1_.

### 2.3. Surface Hardness Test

Vickers hardness indentations were created on the top surface of the specimen using a Vickers hardness tester (MVK-F, AKASHI, Kawasaki, Japan), and the average Vickers hardness was calculated (*n* = 12). The indentations were created using a diamond pyramid indenter with a load of 100 gf and a dwell time of 15 s.

### 2.4. Scanning Electron Microscopy Observation

The S-PRG filler, resin powder, and fractured surface specimens after the bending test were prepared and sputter-coated with Au–Pt. These were examined using scanning electron microscopy (SEM; JEOL JSM-6330F, JEOL, Tokyo) with an accelerating voltage of 5 kV. The shape and size distribution of the S-PRG-1 and S-PRG-3 fillers and the resin powder were also investigated. The fracture specimens were examined to assess adhesion and other defects between the filler and resin matrix after the bending test.

### 2.5. Statistical Analysis

All the recorded statistical data were analyzed using GraphPad Prism version 8.1.2 (GraphPad Software, Inc., La Jolla, CA, USA). Normal distribution and variance equality tests were performed using the Brown–Forsythe test and Bartlett’s test, respectively. The flexural strength, modulus, and hardness data were analyzed using one-way analysis of variance (ANOVA) and Tukey’s multiple comparison tests. The Pearson correlation coefficient was calculated to determine the correlation between the particle sizes and contents and mechanical properties. The significance level was set at 0.05.

## 3. Results

### 3.1. SEM Observation of S-PRG Filler and Resin Powder

**Figure 1:**
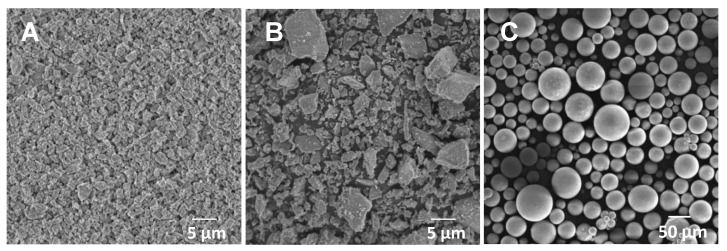
Representative scanning electron microscopy (SEM) images of (**A**) surface pre-reacted glass ionomer (S-PRG)-1, (**B**) S-PRG-3, and (**C**) auto-polymerizing resin (APR) powder.

Figure 1 presents representative SEM images of the S-PRG-1 and S-PRG-3 fillers and the APR powder (polymer). Polygonal particles with various dimensions were observed in the SEM images of the S-PRG fillers. The particle sizes of S-PRG-1 (Figure 1A) were smaller and more uniform compared with those of S-PRG-3. The particle sizes of S-PRG-3 varied from 1.8 μm to 5.2 μm, and the average size was 3 μm. The particle sizes of S-PRG-3 appeared non-uniform despite their regular microstructures (Figure 1B). The particle sizes of the resin powder ranged from 20 μm to 80 μm, and the mean particle size was approximately 50 μm (Figure 1C).

### 3.2. Flexural Strength

**Figure 2:**
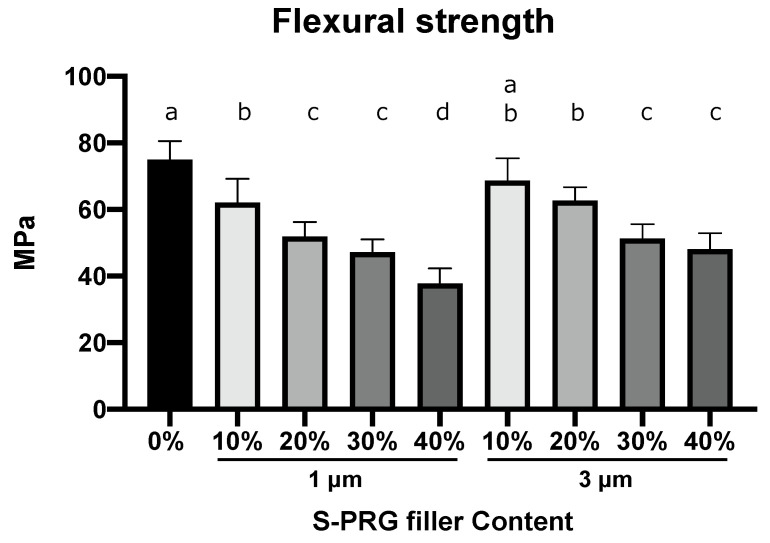
Flexural strength. The bars represent mean values ± standard deviation (*n* = 12). Different letters indicate statistical significance (*p* < 0.05).

Figure 2 presents the flexural strengths of specimens (control, S-PRG-1, and S-PRG-3) with their mean values and standard deviations. The control specimen showed the highest flexural strength of 74.99 ± 5.3 MPa (Table 1). The flexural strength decreased as the amount of S-PRG filler increased. The flexural strengths of the specimens with an S-PRG-1 content of 10% and S-PRG-3 contents of 10% and 20% were significantly higher than those of other S-PRG-containing resins (*p* < 0.05).

### 3.3. Flexural Modulus

**Figure 3:**
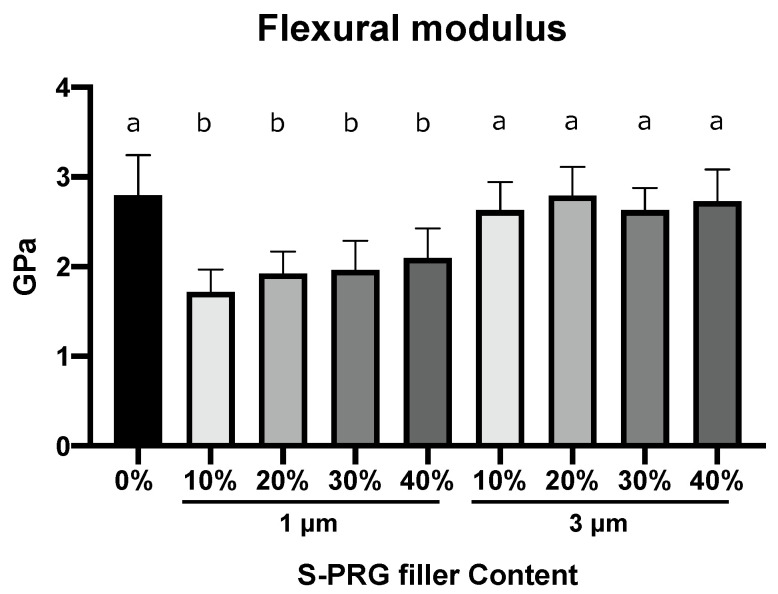
Flexural modulus. The bars represent mean values ± standard deviation (*n* = 12). Different letters indicate statistical significance (*p* < 0.05).

Figure 3 presents the flexural modulus of the specimens (control, S-PRG-1, and S-PRG-3) with their mean values and standard deviations. The control value is 2.80 ± 0.2 GPa (Table 1). The flexural moduli of the specimens of S-PRG-1 at all contents are lower than the control and specimens with the S-PRG-3 at all contents, with a significant difference (*p* < 0.05). A large S-PRG filler size of 3 μm strengthened the flexural modulus more than the filler size of 1 μm. The addition of S-PRG-1 to the APR reduced the flexural modulus.

### 3.4. SEM Observations of Fracture Surfaces after the Bending Test

**Figure 4:**
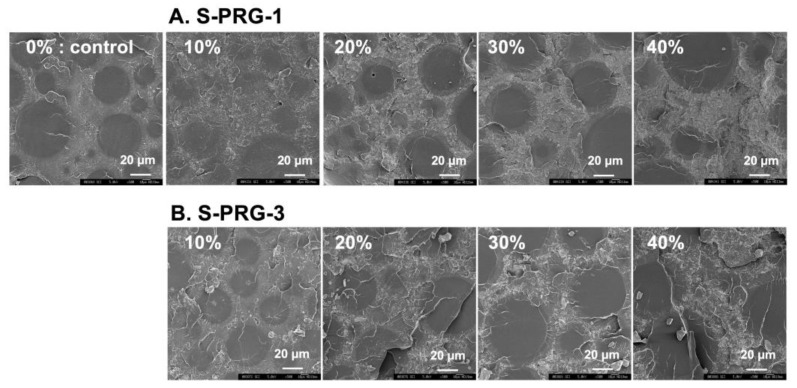
Representative SEM images at the fracture surfaces of the specimens after the three-point bending test. (**A**) S-PRG-1 and (**B**) S-PRG-3 at ×500 magnification.

Figure 4 presents an SEM image of the fractured surface of specimens after the three-point bending test. The control specimen image shows a cured specimen with a monomer and polymerized sphere shape. S-PRG filler particles dispersed and were distributed in the resin matrix, and were well polymerized uniformly with a monomer in the specimens with S-PRG-1 and S-PRG-3. As the S-PRG filler content increases, the matrix becomes dense with the S-PRG filler. The fracture surface of the specimen of S-PRG-1 has many fine fillers compared to those of S-PRG-3. Crack penetration into the polymer loaded by the bending test is observed in the specimen of S-PRG-3 at 20 and 40 wt%.

### 3.5. Surface Hardness

**Figure 5:**
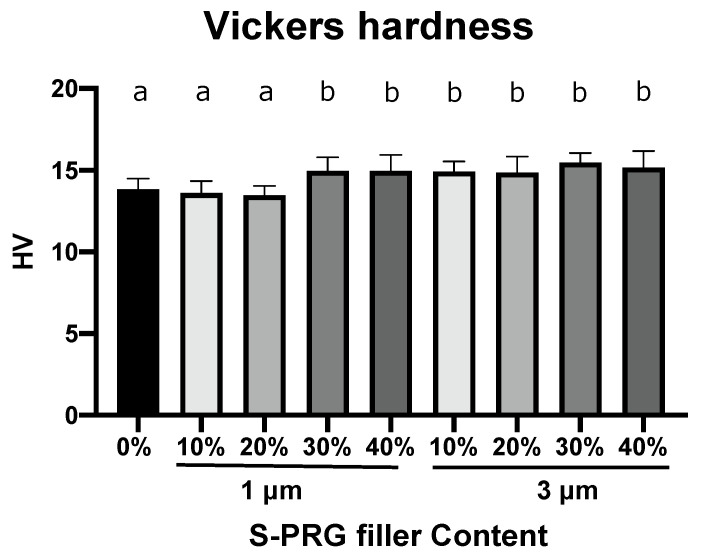
Vickers hardness. The bars represent mean values ± standard deviation (*n* = 12). Different letters indicate statistical significance (*p* < 0.05).

Figure 5 and Table 2 present the Vickers hardness values of the specimens (control, S-PRG-1, and S-PRG-3) with the corresponding mean values and standard deviations. The surface hardness of the control specimen was 13.84 ± 0.6 HV (Table 2). The Vickers hardness value increased with an increase in the filler content and filler size. Specimens with S-PRG-1 contents of 30 and 40 wt%, as well as all S-PRG-3 specimens, exhibited significantly higher Vickers hardness values than the control specimens and specimens with S-PRG-1 contents of 10 and 20 wt% (*p* < 0.05). However, no significant differences in the Vickers hardness values were observed between the control specimen and the specimens with S-PRG-1 contents of 10 and 20 wt% (*p* > 0.05).

### 3.6. Pearson Correlation Test

Since a correlation was found between the mechanical properties of the APR and S-PRG filler content and particle size, a Pearson’s correlation test was performed (Table 3). The S-PRG filler content did not correlate with the flexural modulus. A positive correlation was observed for the Vickers hardness. Interestingly, a strong negative correlation was found for flexural strength. Further, particle size was positively correlated with the mechanical properties. In particular, a strong positive correlation was found for the flexural modulus.

## 4. Discussion

The experiments on incorporating the S-PRG filler in APR confirm good mechanical properties at low S-PRG filler contents. Therefore, the null hypothesis is partially rejected. The flexural strength decreased from the control value of 74.99 MPa (free of S-PRG filler) to the values of specimens containing S-PRG filler following the amount of the S-PRG-1 and S-PRG-3 addition. The experimental resin powders of APR and the S-PRG filler were well dispersed and polymerized with liquid. The specimens became slightly less viscous as the S-PRG filler content increased. No crack penetration was visually observed on the cured specimen surface. The addition of the S-PRG filler did not play a role in strengthening the specimen of APR incorporated with the S-PRG filler prominently overall. However, the flexural strength of the specimens of S-PRG-1 at 10 wt% and S-PRG-3 at 10 wt% and 20 wt% cleared the ISO standard, which stipulates that the flexural strength of APR must be 60 MPa or more [27,28].

Kamijo et al. demonstrated the flexural strength and modulus of experimental denture base resin (heat-polymerizing resin) containing S-PRG-3 at 5, 10, 20, 30, and 40 wt% [29]. The flexural strength at 5, 10, and 20 wt% complied with the requirements of the ISO standard, which states that the flexural strength of denture base resins should be 65 MPa or over [28]. The flexural strength resulting from our study agreed with that of their study when S-PRG-3 was used with a similar content. Although different resins were used in the fracture test, a similar result was obtained: increasing the amount of S-PRG filler decreased the flexural strength. We employed APR, whereas they employed heat-polymerizing PMMA resin. The ISO standard describes that the minimum flexural modulus for denture base resin should not be less than 2.0 GPa [28]. In our study, the flexural modulus of the specimens of S-PRG-1 at 40 wt% and S-PRG-3 at all contents exceeded the ISO standard. Kamijo et al. showed that the flexural modulus of specimens containing S-PRG-3 in all contents was more than 2.0 GPa [29], which is similar to our results. These results demonstrate the validity of our present study. It is essential to determine the optimal percentage of filler that satisfies the need to maintain sufficient strength. Many investigators have tried to modify PMMA polymers to improve their mechanical performance by adding additives such as metal oxides, metal-oxide nanoparticles, polymeric fibers, and glass fibers to enhance their properties for dental applications. Moreover, various factors were important in incorporating fillers into PMMA, such as the filler shape and size, the nature of the matrix bonding, the resins used, and the distribution of the polymer matrix [30]. The filler percentage should be low enough to be securely embedded in the resin. The filler should be small enough to produce uniform mixtures and penetrate between the linear polymer chains [24]. Recent research has focused on nanoparticle fillers. They are widely recognized because they offer advantageous properties due to their composition, shape, size, and ability to improve the original properties of polymers [31,32]. However, while the previous studies have occasionally shown encouraging results, they have often been contradicting. Therefore, adding fillers to auto-polymerizing or heat-polymerizing PMMA resin for reinforcement may increase the fracture risk instead of preventing it [30].

Karci et al. added 1, 3, and 5 wt% of Al_2_O_3_ and TiO_2_ nanoparticles of a 15-nm size to APR and investigated the flexural strength [24]. The flexural strength decreased as the nanoparticles fraction increased. Despite the use of different particles, the overall trend corroborates with that observed in our study. In our investigation, the highest flexural strength values were found at the 10% ratio for S-PRG-3, and the lowest flexural strength values were observed at the 40% ratio for S-PRG-1. Few studies have compared the effect of the filler size on the flexural strength of PMMA resin. Zidan et al. investigated the flexural strength of PMMA resin with ZrO_2_ nanoparticles of sizes between 30 and 100 nm [33]. The flexural strength values at 3 wt% and 5 wt% were 127.1 and 134.9 MPa, respectively. Additionally, Zidan et al. investigated the flexural strength of PMMA resin with ZrO_2_ nanoparticles of sizes between 30 and 60 nm [34]. The flexural strength values at 3 wt% and 5 wt% were 83.5 and 78.7 MPa, respectively. Their results revealed that the flexural strength declined as the filler size reduced, even when the filler content remained the same. In our study, the mean flexural strengths of a specimen of S-PRG-1 and S-PRG-3 at 10 wt% were 62.15 MP and 68.68, respectively. As with earlier studies, the flexural strength of APR decreased as the filler size decreased.

Incidentally, the biological effects of the S-PRG filler have been reported to show outstanding bioactivity owing to the gradual release of six ions. Previous studies have showed that the S-PRG filler releases multiple ions and buffers the demineralization of bovine enamel, indicating its caries-preventive effect [11,12]. S-PRG fillers have been studied for several types of oral pathogens. The S-PRG filler potentially reduces *Candida albicans* adhesion to PMMA resin to decrease denture stomatitis [16]. The ions eluted from the S-PRG filler inhibit the growth of *Streptococcus mutans*, an important microorganism that induces carious lesions associated with the bacterial sugar metabolism, and reduce the formation of biofilms [35]. In recent years, S-PRG fillers with a smaller particle size of 1 μm have been used instead of the conventional size of 3 μm. In a dog study, Mayumi et al. reported that the nano-sized S-PRG filler drastically reduced the inflammatory parameters of the gingival tissue around the premolars during periodontal disease [15]. They used an S-PRG filler with a size of 0.48 μm. This particle size was less than half of the size of 1 μm, which we used. Such biological effectiveness is not reported for the other additives. Hatano et al. reported that denture adhesives containing 1-μm and 3-μm S-PRG fillers were investigated for their antibacterial effects [36]. Their investigation showed that S-PRG-1 had a higher antibacterial effect than the S-PRG-3 denture adhesive. Previous research suggested that the small particle sizes of S-PRG and the high S-PRG content yielded high bioactivity [15,22,36]. This result contradicts the result of mechanical properties. The results from our study suggest that different particle sizes and concentrations of S-PRG fillers affect the mechanical strength of APR. Smaller filler sizes can be used to provide a more detailed understanding of how filler size affects mechanical qualities. The lack of nano-sized S-PRG filler may be a limitation of this study.

Evaluation of denture flexural strength helps to understand how well resins perform under the stress of chewing. The gradual increase in the Vickers hardness of the specimens with the amount of S-PRG-3 in this study may be applied to the random distribution of the S-PRG filler particles in the acrylic matrix. Furthermore, it is likely that S-PRG filler particles might be present, particularly on the surface. A similar tendency was confirmed on the surface of PMMA resin containing nanoparticles [26]. The hardness measurement is an index for understanding resistance to plastic deformation and provides insight into the cutting, finishing, and polishing performance [1]. Denture materials should have sufficient strength to withstand use in the oral cavity and possess a function to undertake continuous oral health maintenance. The ion release is activated by simply wearing dentures containing the S-PRG filler to reduce oral pathogens that induce tooth decay and periodontal diseases [7,37]. This approach explores the development of new prosthodontic PMMA materials that maintain clinical success and long-term durability.

SEM observation of fracture surfaces did not provide clear information as to why the flexural strength of resins containing the S-PRG filler decreases with increasing the S-PRG filler content. However, the S-PRG filler was observed in all specimens and diffused uniformly into the resin matrix, presented in Figure 4. With the observation that the S-PRG filler is simply embedded in the matrix and not chemically bonded to the matrix, the increase in the S-PRG filler may be a factor that does not strengthen the resin matrix. The addition of the S-PRG filler to APR did not resist loading from bending tests. The proper mechanical properties of APR with the S-PRG filler could be improved by several modifications, especially the addition of a nano-S-PRG filler or silane treatment for bonding the PMMA polymer and S-PRG filler. The study of the interfacial bonding between the S-PRG filler and APR is a subject for future research.

In this study, including the S-PRG filler in the PMMA polymer decreased the bendability, which is an important index. A minor limitation of this study is that the minimum concentration is 10%, and no experimental results are available for lower concentrations. Investigating the mechanical properties of APR by fabricating samples with lower concentrations, such as 1.0 wt% and 5.0 wt%, may yield new results. Moreover, it is also necessary to investigate whether a low-concentration S-PRG-containing APR can impart a bioactive effect on dental materials. Materials containing the S-PRG filler may inhibit tooth demineralization and promote remineralization. It may aid in the prevention or cessation of tooth decay. However, PMMA materials must be maintained in the oral cavity for a certain period. When S-PRG filler is added, specific mechanical properties are required. This novel study shows the significance of the size and concentration of the S-PRG filler added to APR and its effects on mechanical strength. This research was limited to the SEM observation of fractured surfaces. An investigation of the adhesive interface and binding mechanism between the APR matrix and S-PRG filler by X-ray diffraction analysis, and Raman and Fourier Transform Infrared spectroscopies will be the subject of future research.

## 5. Conclusions

This study obtained the following results regarding the mechanical properties of APR containing the S-PRG filler.

1. The flexural strength decreased with the increasing S-PRG filler content. The flexural strength and flexural modulus decreased with a smaller S-PRG filler size.
2. The flexural strength of conventional APR incorporated with S-PRG-1 at 10% and S-PRG-3 at 10% and 20% exceeds 60 MPa, passing the requirements of the ISO standards. The flexural modulus and Vickers hardness demonstrate significant properties that could be used in clinical practice.
3. The Pearson’s correlation test determined that the S-PRG filler content and particle size correlated with the mechanical properties of APR. The content of the S-PRG filler did not correlate with the flexural modulus. A positive correlation was observed for Vickers hardness.

## Figures and Tables

**Table 1:** Flexural strength and flexural modulus values of specimens (control, S-PRG-1, and S-PRG-3).

S-PRG Filler Content (wt%)	Particle Size (μm)	Flexural Strength (MPa)	Flexural Modulus (GPa)
Control (0)	Control (0)	74.99 ± 5.3 ^a^	2.80 ± 0.2 ^d^
10	1	62.15 ± 6.6 ^b^	1.72 ± 0.2 ^e^
20	1	51.87 ± 4.2 ^c^	1.93 ± 0.2 ^e^
30	1	47.15 ± 3.7 ^c^	1.96 ± 0.3 ^e^
40	1	37.80 ± 4.3 ^d^	2.10 ± 0.3 ^e^
10	3	68.68 ± 6.4 ^ab^	2.63 ± 0.3 ^d^
20	3	62.70 ± 3.8 ^b^	2.79 ± 0.3 ^d^
30	3	51.28 ± 4.2 ^c^	2.63 ± 0.2 ^d^
40	3	48.05 ± 4.7 ^c^	2.73 ± 0.3 ^d^

Data are presented as mean values ± standard deviation (*n* = 12). One-way ANOVA for the nine groups yielded *p* < 0.05. Different letters indicate statistically significant differences, based on Tukey honestly significant difference (HSD) tests for multiple comparisons at *p* < 0.05.

**Table 2:** Vickers Hardness values of the specimens (control, S-PRG-1, and S-PRG-3).

S-PRG Filler Content (wt%)	Particle Size (μm)	Vickers Hardness (HV)
Control (0)	Control (0)	13.84 ± 0.6 ^a^
10	1	13.61 ± 0.6 ^a^
20	1	13.48 ± 0.5 ^a^
30	1	14.95 ± 0.8 ^b^
40	1	14.97 ± 0.9 ^b^
10	3	14.92 ± 0.6 ^b^
20	3	14.86 ± 0.9 ^b^
30	3	15.48 ± 0.6 ^b^
40	3	15.18 ± 1.0 ^b^

Data are presented as mean values ± standard deviation (*n* = 12). One-way ANOVA for the nine groups yielded *p* < 0.05. Different letters indicate statistically significant differences, based on Tukey HSD tests for multiple comparisons at *p* < 0.05.

**Table 3:** Results of the Pearson correlation test between S-PRG filler content or particle size and mechanical properties using calculated coefficient *r* and *p* values.

		S-PRG Filler Content	Particle Size
Flexural strength	r value	−0.894	0.420
	*p* value	0.003	0.300
Flexural modulus	r value	0.185	0.992
	*p* value	0.660	0.001
Vickers hardness	r value	0.570	0.628
	*p* value	0.139	0.095

## Data Availability

The data used to support this study are available from the corresponding author upon request.
